# Improving pain treatment with a smartphone app: study protocol for a randomized controlled trial

**DOI:** 10.1186/s13063-018-2539-1

**Published:** 2018-02-27

**Authors:** Carlos Suso-Ribera, Ángela Mesas, Javier Medel, Anna Server, Esther Márquez, Diana Castilla, Irene Zaragozá, Azucena García-Palacios

**Affiliations:** 1 Department of Basic and Clinical Psychology and Psychobiology, Jaume I University, Av. de Vicent Sos Baynat, s/n, 12071 Castellon de la Plana, Spain; 2 Pain Clinic, Vall d’Hebron Hospital, Barcelona, Spain; 3 CIBER of Physiopathology of Obesity and Nutrition CIBERobn, CB06/03 Instituto de Salud Carlos III, Madrid, Spain

**Keywords:** Chronic pain, Smartphone app, Ecological momentary assessment, Telemonitoring, Randomized controlled trial

## Abstract

**Background:**

Chronic pain has become a major health problem across the world, especially in older adults. Unfortunately, the effectiveness of medical interventions is modest. Some have argued that assessment strategies should be improved if the impact of medical interventions is to be improved. Ecological momentary assessment using smartphones is now considered the gold standard in monitoring in health settings, including chronic pain. However, to the best of our knowledge, there is no randomized controlled trial to show that telemonitoring using a smartphone app can indeed improve the effectiveness of medical treatments in adults with chronic pain. The goal of this study will be to explore the effects of using a smartphone app for telemonitoring adults with chronic pain.

**Methods:**

The study will be a randomized controlled trial with three groups: treatment as usual (TAU), TAU+app, and TAU+app+alarms. All groups will receive the adequate treatment for their pain, which will be prescribed the first day of study according to clinical guidelines. Assessment in the TAU group will be the usual at the Pain Clinic, that is, a paper-and-pencil evaluation at the onset of treatment (beginning of study) and at follow up (end of study, 30 days later). The other two groups (TAU+app and TAU+app+alarms) will be assessed daily using Pain Monitor, a smartphone app developed by our multidisciplinary team. Telemonitoring will only be made in the TAU+app+alarms group. For this group, physicians at the Pain Clinic may decide to adjust pain treatment in response to alarms. Telemonitoring is not the usual practice at the Pain Clinic and will not occur in the other two groups (TAU and TAU+app), so no changes in treatment are expected in these groups after the first appointment. The total sample size will be 150, with 50 patients in each group. The assessment protocol will be the same in all groups and will include pain intensity and side effects of the medication (primary outcomes), together with several pain-related variables like pain interference, activity level, use of rescue medication, pain catastrophizing, and pain acceptance, among others.

**Discussion:**

We believe that the present trial has important clinical implications. We think that telemonitoring using ecological momentary assessment is crucial to improve current interventions for pain. The armamentarium of available treatments for pain is large, so physicians can turn to different treatments or dosages in the presence of an undesired event. The use of the app for telemonitoring can allow for this rapid detection of unwanted events, thus improving patient safety (i.e., withdrawal of treatment causing side effects) and augmenting treatment effectiveness (i.e., changing an ineffective treatment or dosage). In a time when smartphones are a mainstream technology, we should take advantage of them in the promotion of health care.

**Trial registration:**

ClinicalTrials.gov, NCT03247725. Registered on 25 July 2017.

**Electronic supplementary material:**

The online version of this article (10.1186/s13063-018-2539-1) contains supplementary material, which is available to authorized users.

## Background

Pain is “a distressing experience associated with actual or potential tissue damage with sensory, emotional, cognitive and emotional components” [1]. Pain is sometimes short in duration and disappears as tissues heal. When this happens pain is considered to be acute. Unfortunately, pain can also persist over long periods of time and become chronic. A cutoff of between 3 and 6 months is, in the absence of other criteria (i.e., normal healing period of an injury), the one proposed to differentiate acute from chronic pain [2].

Chronic pain has become a major public health problem due to its high prevalence. This disease is estimated to affect 20–30% of the adult population across the world [3–6]. Projections of future prevalence of chronic pain are not more encouraging. With the rise of life expectancy, the age distribution of the population is changing towards the elderly [7]. This is likely to have important implications for chronic pain as its prevalence increases dramatically with age. For example, a study conducted in Spain showed that the percentage of individuals with chronic pain increases to 73.5% in people over 65 years old [8].

Medical interventions are the first-line interventions recommended in recent guidelines for chronic pain [9–11]. Unfortunately, support for their effectiveness is only modest [12]. In fact, the most potent drugs (i.e., opioids) only seem to reduce pain by 30–40% in less than half of the patients [13] and a number of patients experience significant side effects [14]. Likewise, surgery (i.e., spinal cord stimulation and spinal fusion) only appears to provide partial reduction in pain intensity in a subset of patients, and complications after surgery are frequent [15, 16].

Some have argued that the limited efficacy of medical treatment for pain might be partly due to deficits in assessment methodology [12, 17]. For instance, existing randomized controlled trials (RCTs) in chronic pain tend to include a reduced number of measurement points. This is a problem because pain-related variables, such as pain intensity and fatigue, can vary across and within days [18]. Also, when continuous assessment has been included, studies have mostly relied on paper diaries or recalled information, which might be problematic because of fake data entry, disagreement between momentary and recalled pain data, and, importantly for the present study, inability to be used for telemonitoring [19, 20]. Thus, ecological momentary assessment (EMA) using electronic diaries has now been claimed to be the “gold standard” in healthcare monitoring [21]. EMA, which involves real-time, repeated assessment, has been demonstrated to minimize recall bias and maximize ecological validity [22]. Additionally, the use of electronic diaries, as opposed to traditional paper diaries, enhances compliance, reduces errors in data entry, and allows for telemonitoring [23, 24].

An example of how assessment can be responsible for deficits in medical treatment for pain is also given in the context of our Pain Clinic at the Vall d’Hebron Hospital, but may be applicable to other pain clinics worldwide. Patients at our Pain Clinic tend to be assessed for pain intensity, side effects of the medication, and other pain-related variables during face-to-face appointments only. When patients have a concern between appointments (i.e., pain does not decrease or they are experiencing a side effect of the medication), it is the patient’s decision to take action. From our past experience, they either go to emergency services, to their general practitioner, they try to contact the Pain Clinic by phone, or they do nothing and wait until the following appointment at the Pain Clinic. This procedure is problematic. First, emergency services and general practitioners are not specialized in pain treatment and have limited treatment knowledge and options when compared to physicians at the Pain Clinic. The phone-call procedure is inefficient, as it is very costly (i.e., it requires that someone is available at all times at the Pain Clinic to answer to those calls) and does not guarantee that patients’ concerns are clinically relevant nor that all patients with clinically relevant events call the Pain Clinic, which is a threat to patient safety.

The use of smartphone applications in health settings has been boosted in recent years, especially as an alternative or adjuvant to on site, face-to-face treatments [25–27]. However, to the best of our knowledge there is no RCT in pain settings showing that the use of an electronic diary for EMA increases the effectiveness of medical treatment. The goal of the present study is to explore whether the inclusion of a smartphone app for daily telemonitoring of chronic pain improves the effectiveness of medical treatment in patients with pain. We expect that telemonitoring will contribute to more patient-centered care, as revealed in previous research [17], thus resulting in reduced pain intensity ratings and decreased duration and frequency of side effects of the medication. To ensure that physician telemonitoring and not the use of the app itself is responsible for increased treatment effectiveness, a group of patients will use the app daily without telemonitoring (no alarms in the presence of undesired events) and compared against a group using the app with telemonitoring. A third group will follow the usual measurement without the app.

## Methods/design

### Design

This superiority, randomized controlled trial was approved by the Ethical Review Board at the Vall d’Hebron Hospital in Barcelona (June 25, 2017) and registered in the clinicaltrials.gov registry on July 25, 2017 (NCT03247725). A Standard Protocol Items: Recommendations for Interventional Trials (SPIRIT) checklist can be found in Additional file 1.

**Fig. 1:**
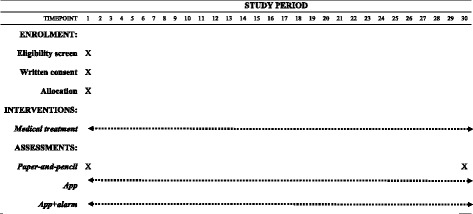
Schedule of enrolment, interventions, and assessments. All patients received the usual treatment for their pain irrespective of their group allocation

Once eligibility is ensured, patients will be randomly allocated to one of three conditions: (1) treatment as usual (TAU); (2) TAU + daily assessment using the Pain Monitor app; or (3) TAU + daily assessment using the Pain Monitor app with alarms. Assignment will be made by an independent researcher using an online randomization tool. Neither patients nor physicians in charge of treatment will be blind to allocation. The SPIRIT flowchart is shown in Fig. 1.

### Eligibility criteria

#### The eligibility criteria are:

a.The patient is over 18 years of age

b.The patient does not present with psychological and/or cognitive alterations or problems with language that make their participation difficult

c.The patient has the physical ability to use the application

d.The patient voluntarily wants to participate and signs the informed consent form (Additional file 2)

e.The patient has a mobile phone with an Android operating system

### Procedure

The study will be advertised by physicians working at the pain unit of the Vall d’Hebron Hospital in Barcelona. All consecutive patients meeting the eligibility criteria of (a) age and (b) ability to participate will be introduced to the study. An information sheet will be provided and if the patient agrees to participate in the study, the inclusion criteria (c) and (e) will be explored. Participants meeting all previous inclusion criteria will be asked to sign the informed consent form (criterion d) and will be assigned to a previously randomized study condition.

### Sample

The participants will be 150 patients with chronic pain, attending the pain clinic at the Vall d’Hebron Hospital in Spain. There will be no exclusion criterion in terms of existing treatment for pain at study onset or changes in treatment during the study, so that the sample will be representative of patients treated at the pain clinic. However, this information will be collected in all participants regardless of the assigned condition.

Participants in the TAU condition will be identified using a unique alphanumeric code. Their responses will not contain any identifying information. The database provided by the app (app and app+alarm condition) will also be completely anonymized, since the system will not store any personal information. The app will only collect the international mobile equipment identity (IMEI), which is a reference unique to each mobile phone. To ensure confidentiality, the storage of participants’ IMEI will be external to the system. The file containing the link between non-identifying codes (IMEI or alphanumeric code) and patients’ identifying information (medical registry number) will be stored locally in a separate computer. Data collection and storage will follow Spanish law and data protection rules (“Ley Orgánica 15/1999, de 13 de diciembre, de Protección de Datos de carácter personal”, “RD 1720/2007, de 21 de diciembre, por el que se aprueba el reglamento de desarrollo de la LOPD (RLOPD)”, and “Ley 34/2002, de 11 de julio de Servicios de la Sociedad de la Información y de comercio electrónico”).

### Pain monitor

Content in the app was developed by a multidisciplinary team of psychologists, physicians, and nurses from the Pain Clinic of the Vall d’Hebron Hospital and the Labpsitec group of the Jaume I University via consensus after a set of meetings. Assessed constructs were selected according to existing guidelines on pain measurement [12, 28–30] and included demographic information, pain characteristics (location, duration, intensity, and neuropathic symptoms), treatment for pain, use of rescue medication, side effects, adherence to treatment, pain interference, fatigue, mood (depression, anxiety, anger, and happiness), perceived health status, pain catastrophizing, pain acceptance and willingness, coping, fear/avoidance of pain, activity level, and satisfaction with treatment. The assessment protocol in the app was validated against well-established measures in a previous study with 38 patients attending the Pain Clinic of the Vall d’Hebron Hospital in Barcelona. These include the Brief Pain Inventory for pain intensity and pain interference [31], the Profile of Mood States for fatigue and mood [32], the Hospital Anxiety and Depression Scale for depression and anxiety [33], the Beck Depression Inventory-II for depression [34], the Short Form 12 for perceived health status [35], the Pain Catastrophizing Scale for pain catastrophizing [36], the Chronic Pain Acceptance Questionnaire for pain acceptance and willingness [37], the Fear-Avoidance Beliefs Questionnaire for fear/avoidance of pain [38], the Chronic Pain Coping Inventory-42 [39] and the Coping Strategies Questionnaire [40] for coping, and the Roland Morris Disability Questionnaire for activity level despite pain [41]. The list of side effects was created considering the most frequent adverse effects of pain medication [42–44]. All the questions in the app can be found in Additional file 3.

The primary measures will be pain intensity and side effects of the medication, which are expected to be lower in the app+alarm condition. Secondary measures will include the remaining variables, including pain interference, mood, use of rescue medication, satisfaction with treatment, perceived health status, activity level, fear of pain, pain acceptance, and pain catastrophizing. Again, all of these are hypothesized to show a greater improvement in the app+alarm condition.

**Fig. 2:**
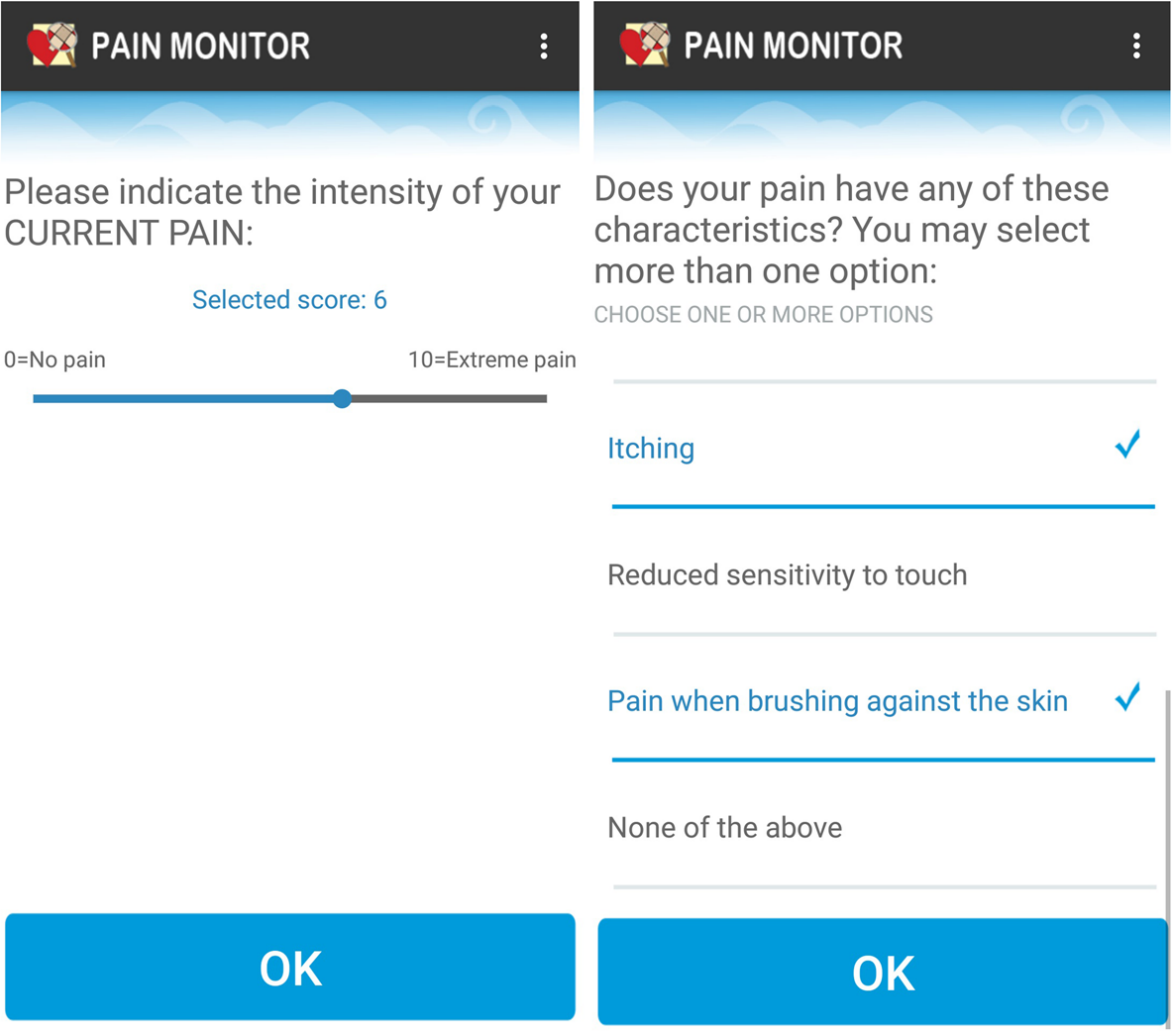
Example of two app items

Two examples of app items are presented in Fig. 2.

### Interventions

All participants will receive the usual medical treatment at the pain unit, following existing guidelines for pain management [42, 45]. During the first appointment (beginning of study), physicians will propose a treatment plan for each patient according to the aforementioned guidelines. All patients will have a follow-up appointment 1 month after the onset of treatment (end of study).

As shown before, response to undesired clinical events (i.e., pain is not decreasing or patient experiences side effects of a medication) varies widely across patients and depends on patients’ own judgment. Some patients go to the emergency service or to the general practitioner. Others try to contact physicians at the Pain Clinic by phone, which is not always possible, while a subset of patients just tolerate the symptoms and wait until the next appointment. In all cases, the “alarm” and the response to it is based on the patient’s criterion, which, as shown before, is problematic. In the present study, this procedure will be representative of patients in the TAU and TAU+app conditions. In contrast, in the TAU+app+alarm group, clinically relevant events will be determined by physicians in the app and alarms will be received daily to offer a rapid response (i.e., phone patients and make changes in the medication, which can be collected from their general practitioner).

As indicated above, changes in pain medication may not only occur in the alarm group, but also in the other two conditions (i.e., if patients attend the emergency service, their general practitioner, or if they call the Pain Clinic), so this will be controlled at the end of the study by looking at the hospital database.

### Assessment plan

For all conditions, patients will be asked to respond to the pre-treatment assessment during the beginning of the study appointment, before the treatment plan is proposed. Patients assigned to the app or app+alarm conditions will be asked to download and complete the pre-treatment assessment in the app. The physician will assist in this first app use. In this pre-treatment assessment with the app, patients have to report on demographic and pain information. Then, participants are asked to answer to a morning and an evening assessment daily for 30 consecutive days. Some items are asked twice a day (i.e., pain intensity, fatigue, and mood), while others are only asked once, in the morning (i.e., interference of pain on past night sleep) or in the evening (i.e., interference of pain on leisure, activity level during the day, and side effects). At the end of the study (day 30 of app use), information on satisfaction with treatment and pain characteristics (i.e., location and neuropathic symptoms) is collected. The app uses a push system to inform patients when to respond. Default hours are set (10 am and 7 pm), but they can be changed with 2-h flexibility.

As usual, patients in the TAU condition will be assessed only twice, at the beginning of the study and in the follow-up appointment (end of study). Despite the traditional assessment at the Pain Clinic being limited to assessment of pain characteristics (duration, location, intensity, and neuropathic symptoms), we will include the whole assessment protocol used in the app to make results comparable. As usual, this assessment will be made in a paper-and-pencil format.

### Calculations and analyses

Considering previous studies on the complications of pain treatments [14, 46], we expect that their rapid detection using alarms might yield moderate between-group differences in primary measures, that is, pain intensity and frequency and duration of side effects (Cohen’s *d* = 0.5). Despite being only speculative, it is also possible that patients allocated to the app without alarm will perform better than those allocated to TAU, if they also perceive that they are being telemonitored even though they are not. However, we expect this effect to be small. Considering 80% power, we will need 50 participants in each group to perform the analyses. Power calculations were made with G*Power [47]. Data will be analyzed using the intention-to-treat principle and a mixed-model approach [48]. Changes in secondary measures will also be explored, although they are expected to be small because they are not the main target of medical intervention in the present study. Analyses will be performed separately by the lead researcher, C.S.R., and an independent researcher. Interim analyses will not be performed because no harm is expected from adding the app to the usual treatment. The final dataset and the statistical code will be publically accessible to all researchers under request, excluding any personal information from the participants. Changes to eligibility and other protocol modifications, if any, will be discussed and communicated to relevant parties (Ethical Review Board, clinicians, participants, and journals).

## Discussion

Treatment of pain can be complex due to individual differences in response to interventions, the vast repertoire of available treatments, side effects associated with certain interventions, and the challenges of choosing the right dosage for a given patient [42–44, 49]. Thus, personalized treatments are urgently required. EMA has been argued to be a prerequisite to achieve this improvement in the effectiveness of treatments for chronic pain [17, 50]. There are important problems associated with the use of paper diaries for assessment of EMA, including recall bias and limited naturalistic value [51, 52]. The use of an app is a promising way of dealing with limitations of paper diaries, and is an easy tool for use in telemonitoring.

In the present study protocol, we describe a RCT designed to test the utility of Pain Monitor, an app for daily assessment of adults with chronic pain. The study aims to explore whether the use of the app results in improved pain treatment by adding a telemonitoring tool, that is, alarms in the presence of undesired events like side effects of the medication, lack of response to treatment, or excessive use of rescue medication. To the best of our knowledge this is the first study to evaluate the impact of a smartphone app for telemonitoring in pain settings and should reveal whether daily monitoring indeed improves treatment effectiveness.

The present study has some limitations. Blinding participants and healthcare professionals to allocation was not feasible for ethical reasons. Also, the app is still not available for the iOS operating system, so a subset of patients will not be able to participate in the study. We expect this number to be small because, in Spain, 90% of smartphones use the Android operating system [53]. If, indeed, results suggest that the use of the app (with or without alarms) leads to improved treatment, Pain Monitor will be developed for the iPhone operating system.

Despite these limitations, if telemonitoring with Pain Monitor leads to the hypothesized results, this will have important clinical implications. Specifically, we expect that daily use of Pain Monitor with alarms will lead to more effective and safer treatments, thanks to the rapid detection of non-response to the intervention prescribed for pain and side effects of the medication. Additionally, the use of Pain Monitor might also be useful to detect changes in other pain-related variables, such as pain interference, activity level, or psychological constructs like pain catastrophizing or pain acceptance, which are key elements in the multidisciplinary treatment of chronic pain [54]. If the results support the use of Pain Monitor, the findings will be communicated to the relevant groups, including the Hospital Board Committee, healthcare professionals, and patients. Also, results will be disseminated to the wider public via scientific publication.

### Trial status

The trial is currently recruiting. Recruitment started in September 2017 and will continue until 150 participants have been included.

## Additional files

Additional file 1: Standard Protocol Items: Recommendations for Interventional Trials 2013 Checklist: recommended items to address in a clinical trial protocol and related documents. (DOC 119 kb)

Additional file 2: Informed consent form. (DOCX 116 kb)

Additional file 3: Items in the Pain Monitor app. (DOCX 27 kb)

## Abbreviations

EMA: Ecological momentary assessment
RCT: Randomized controlled trial
SPIRIT: Standard Protocol Items: Recommendations for Interventional Trials
TAU: Treatment as usual
