# Supplementary material for: Improving pain treatment with a smartphone app: study protocol for a randomized controlled trial
Source: Trials. 2018 Feb 27;19:145. doi: 10.1186/s13063-018-2539-1 (PMC6389085; doi:10.1186/s13063-018-2539-1)
Supplement: Supplementary file 2 — Informed consent form. (DOCX 116 kb) [file 13063_2018_2539_MOESM2_ESM.docx]

**Additional file 2: Informed consent form**

Study title: Assessing the effectiveness of the treatment for pain. Pilot study at the Pain Unit of Vall d'Hebron Hospital

I (given name and surname) _______________________________

I have read the information sheet that has been given to me.

I was able to ask questions about the study.

I have spoken with Dr. ………………………………………………...

I understand that my participation is voluntary and that my data cannot be associated to an identified or identifiable person because the information that identifies that person has been replaced by a code.

I understand that I can withdraw from the study:

1. Whenever I want

2. Without having to give further explanations

3. Without this having an impact on my medical care

I freely give my consent to participate in the study.

Date and signature of the participant Date and signature of the physician
