# Supplementary material for: Improving pain treatment with a smartphone app: study protocol for a randomized controlled trial
Source: Trials. 2018 Feb 27;19:145. doi: 10.1186/s13063-018-2539-1 (PMC6389085; doi:10.1186/s13063-018-2539-1)
Supplement: Supplementary file 3 — Items in the Pain Monitor app. (DOCX 27 kb) [file 13063_2018_2539_MOESM3_ESM.docx]

**Additional file 3: Items in the Pain Monitor app**

*Items assessed once, the first day of app use:*

1. Please indicate your date of birth (DD/MM/YYYY)
2. Please indicate your gender:
   1. Male
   2. Female
3. Please indicate your type of pain. You may select more than one option:
   1. Fibromyalgia
   2. Low back pain
   3. Cervical pain
   4. Rheumatoid arthritis
   5. Osteoarthritis; Headache
   6. Neuropathic pain
   7. Cancer pain
   8. None of the above
4. If you selected “None of the above” please indicate your type of pain. Otherwise, leave this question blank. Press OK to continue.
5. Please indicate the location where your pain is more intense:
   1. Head
   2. Shoulder
   3. Neck
   4. High back
   5. Lower back
   6. Arm
   7. Elbow
   8. Wrist
   9. Hand
   10. Abdomen
   11. Chest
   12. Buttock
   13. Hip
   14. Leg
   15. Knee
   16. Foot
   17. Whole body
   18. Somewhere not listed
6. Who is currently treating your pain? You may select more than one option:
   1. General practitioner
   2. Rheumatologist
   3. Orthopedic specialist
   4. Rehabilitation physician
   5. Psychiatrist
   6. Pain Unit
   7. Neurosurgeon
   8. Neurologist
   9. Oncologist
   10. Another professional.
7. When did your current pain start?
   1. Less than one year ago
   2. Between 1 and 5 years ago
   3. Between 5 and 10 years ago
   4. More than 10 years ago
8. What is your current treatment for pain? You may select more than one option:
   1. Physiotherapy
   2. Pharmacotherapy
   3. Infiltrations
   4. Psychological treatment
   5. Natural / alternative treatments
   6. My pain is not being treated
9. Did you start a new treatment for pain in the last month?
   1. Yes
   2. No
10. Please select the treatment/s you started in the last month. You may select more than one option:
    1. Physiotherapy
    2. Pharmacotherapy
    3. Infiltrations
    4. Psychological treatment
    5. Natural / alternative treatments
    6. I have not started a new treatment
11. What is your marital status?
    1. Single
    2. Married
    3. In a relationship
    4. Divorced
    5. Separated
    6. Widowed
12. What is your job status?
    1. Active worker
    2. Sick leave
    3. Permanent disability
    4. Unemployed
    5. Homemaker
    6. Retired
    7. Student
13. What is the highest level of education you have completed?
    1. No studies
    2. Less than high school
    3. High school graduate
    4. Technical training
    5. University degree
14. Do you currently have a diagnosis of depression by a physician or a psychologist?
    1. Yes
    2. No
15. Do you currently have a diagnosis of anxiety by a physician or a psychologist?
    1. Yes
    2. No

*Items assessed twice a day and in the event of acute pain episodes:*

1. Please indicate the intensity of your CURRENT PAIN:

0 No pain ---------10 Extreme pain

1. Please indicate the intensity of your CURRENT FATIGUE:

0 No fatigue ---------10 Extreme fatigue

1. Please indicate the intensity of your CURRENT HAPPINESS:

0 No happiness -------10 Extremely happy

1. Please indicate the intensity of your CURRENT SADNESS:

0 No sadness -------- 10 Extremely sad

1. Please indicate the intensity of your CURRENT ANXIETY:

0 No anxiety ------- 10 Extremely anxious

1. Please indicate the intensity of your CURRENT ANGER:

0 No anger ------- 10 Extremely angry

1. Does your pain have any of these characteristics? You may select more than one option:
   1. Burning
   2. Painful cold
   3. Electric shocks
   4. Tingling
   5. Pins and needles
   6. Numbness
   7. Itching
   8. Reduced sensitivity to touch
   9. Pain when brushing against the skin
   10. None of the above

*Items assessed in the morning:*

1. In general, your HEALTH is:
2. Very poor
3. Poor
4. Average
5. Good
6. Very good
7. Did your PAIN interfere with the quality of your SLEEP LAST NIGHT?

0 No interference ------- 10 Maximum interference

1. Indicate your degree of agreement with the following sentence: With my current pain, I should not do my usual job (it includes housework and work outside the home).
2. Strongly disagree
3. Disagree
4. Neither agree nor disagree
5. Agree
6. Strongly agree
7. Indicate your degree of agreement with the following sentence: Experiencing pain is terrible and I feel that pain is stronger than me.
8. Strongly disagree
9. Disagree
10. Neither agree nor disagree
11. Agree
12. Strongly agree
13. Indicate your degree of agreement with the following sentence: I need some control over pain before I can make serious plans.
14. Strongly disagree
15. Disagree
16. Neither agree nor disagree
17. Agree
18. Strongly agree
19. Indicate your degree of agreement with the following sentence: Physical activity aggravates my pain.
20. Strongly disagree
21. Disagree
22. Neither agree nor disagree
23. Agree
24. Strongly agree
25. Indicate your degree of agreement with the following sentence: I am living a rewarding life despite my pain.
26. Strongly disagree
27. Disagree
28. Neither agree nor disagree
29. Agree
30. Strongly agree

*Items assessed in the evening:*

1. Did your PAIN interfere with your ability to perform your USUAL WORK or HOUSEWORK TODAY?

0 No interference ------- 10 Maximum interference

1. Did your PAIN interfere with your LEISURE ACTIVITIES TODAY?

0 No interference ------- 10 Maximum interference

1. Did your PAIN interfere with your SOCIAL INTERACTIONS TODAY?

0 No interference ------- 10 Maximum interference

1. Which STRATEGY did you use to COPE WITH YOUR PAIN TODAY? You may select more than one option:
2. Inactivity / rest
3. Relaxation exercise
4. Speak with someone
5. Physical Activity / Stretching
6. Self-statements to persist in a task
7. Do something to feel positive emotions
8. Ignore the pain/distract
9. Pray for the pain to disappear
10. Indicate your degree of agreement with the following sentence: I fear that the pain will get worse.
11. Strongly disagree
12. Disagree
13. Neither agree nor disagree
14. Agree
15. Strongly agree
16. Indicate your degree of agreement with the following sentence: Today I could not keep my pain out of my mind.
17. Strongly disagree
18. Disagree
19. Neither agree nor disagree
20. Agree
21. Strongly agree
22. Please rate your degree of activity TODAY:

0%= Completely inactive -100%= Completely active.

1. In which area have you been more active today? You may select more than one option:
2. Work
3. Family
4. Couple
5. Friends
6. Leisure
7. Physical activity
8. Other.
9. Did you take a rescue medication TODAY (i.e., medication you only use in the event of acute pain)?
   1. Yes
   2. No
10. Did you experience any of these symptoms TODAY? You may select more than one option:
    1. Nausea
    2. Vomiting
    3. Tachycardia
    4. Constipation
    5. Drowsiness / sedation
    6. Blurred vision
    7. Dry mouth
    8. Headache
    9. None of the above
11. Did you experience any of these symptoms TODAY? You may select more than one option:
    1. Dizziness
    2. Itching
    3. Diarrhea
    4. Gait instability
    5. Excessive sweating
    6. Fever
    7. Urine retention
    8. Facial redness
    9. A different symptom
    10. None of the above
12. Did you take your prescribed medication TODAY?
13. Yes
14. No, but I will do it later
15. No and I do not plan to take it
16. I haven't been prescribed a pain medication
17. How many times did you take a rescue medication TODAY?
18. 0
19. 1
20. 2
21. 3
22. 4
23. 5
24. 6
25. 7
26. 8
27. 9
28. 10
29. More than 10

*Items assessed the last day of app use:*

1. With respect to the beginning of treatment, how are you feeling NOW?
2. Much worse
3. Somewhat worse
4. The same
5. Somewhat better
6. Much better
7. Have you experienced any negative life event in the PAST MONTH?
   1. No
   2. Yes, but it did not affect me at all
   3. Yes, but it did not affect me much
   4. Yes and it had quite an effect on me
   5. Yes and it affected me a lot
8. If you experienced a major negative life event in the last month, please indicate its characteristics using the list below. You may select more than one option:
   1. Death of a close person
   2. Job problem
   3. Relationship problem
   4. Economic problem
   5. Health problem
   6. Family problem
   7. An event not listed above
   8. I have not experienced any major negative event this month
9. Please indicate the location where your pain is more intense:
   1. Head
   2. Shoulder
   3. Neck
   4. High back
   5. Lower back
   6. Arm
   7. Elbow
   8. Wrist
   9. Hand
   10. Abdomen
   11. Chest
   12. Buttock
   13. Hip
   14. Leg
   15. Knee
   16. Foot
   17. Whole body
   18. Somewhere not listed
10. What is your current treatment for pain? You may select more than one option:
    1. Physiotherapy
    2. Pharmacotherapy
    3. Infiltrations
    4. Psychological treatment
    5. Natural / alternative treatments
    6. My pain is not being treated
11. Did you start a new treatment for pain in the last month?
    1. Yes
    2. No
12. Please select the treatment/s you started in the last month. You may select more than one option:
    1. Physiotherapy
    2. Pharmacotherapy
    3. Infiltrations
    4. Psychological treatment
    5. Natural / alternative treatments
    6. I have not started a new treatment
13. What is your marital status?
    1. Single
    2. Married
    3. In a relationship
    4. Divorced
    5. Separated
    6. Widowed
14. What is your job status?
    1. Active worker
    2. Sick leave
    3. Permanent disability
    4. Unemployed
    5. Homemaker
    6. Retired
    7. Student
15. Do you currently have a diagnosis of depression by a physician or a psychologist?
    1. Yes
    2. No
16. Do you currently have a diagnosis of anxiety by a physician or a psychologist?
    1. Yes
    2. No
